# Autosomal dominant myopathy caused by a novel *ISCU* variant

**DOI:** 10.3389/fgene.2025.1605440

**Published:** 2025-06-02

**Authors:** Joanna M. Rusecka, Camilla Ceccatelli Berti, Dominika Szczęśniak, Małgorzata Bednarska-Makaruk, Magdalena Mroczek, Magdalena M. Kacprzak, Agnieszka Sobczyńska-Tomaszewska, Paola Goffrini

**Affiliations:** ^1^ MedGen Medical Center, Warsaw, Poland; ^2^ Maria Skłodowska-Curie Medical Academy, Warsaw, Poland; ^3^ Department of Chemistry, Life Sciences and Environmental Sustainability, University of Parma, Parma, Italy; ^4^ Department of Genetics, Institute of Psychiatry and Neurology, Warsaw, Poland; ^5^ Department of Biomedicine, University Hospital Basel and University of Basel, Basel, Switzerland

**Keywords:** mitochondrial myopathy, mitochondria, ISCU, yeast model, WES

## Abstract

Hereditary myopathy with lactic acidosis due to Iron-Sulfur Cluster Assembly Enzyme (ISCU) deficiency is a rare disorder of energy metabolism characterized clinically by myopathy with exercise intolerance, and biochemically by deficiencies of skeletal muscle mitochondrial respiratory chain enzymes. ISCU protein plays an important role in iron-sulphur clusters (Fe-S) assembly and is therefore essential for the activity of mitochondrial Fe-S proteins such as succinate dehydrogenase and aconitase. Recessive hypomorphic *ISCU* alleles have been associated with hereditary myopathy with lactic acidosis, also known as Swedish-type myopathy. To date, only one heterozygous dominant variant (c.287G>T, p.Gly96Val) in the ISCU gene has been reported as pathogenic. Functional studies have shown that this variant has a detrimental, dominant effect on activity of Fe-S-dependent enzymes. Whole exome sequencing performed in an adult female patient with progressive muscle weakness led to the identification of a novel heterozygous variant c.399del (p.Val134Ter) in the ISCU gene. This variant is localized in the functional IscU_like domain of the ISCU protein, with bioinformatics prediction of damaging effects on protein function. Moreover, the same variant was also found in a few family members, who present signs of myopathy. This novel variant segregates with the disease and results in a phenotype reminiscent of the recessive disease previously reported. Yeast *Saccharomyces cerevisiae* is a widely used tool able to assess the impact of the VUS in a quick and efficient way, therefore functional studies were performed on this model system. The results obtained not only confirm the pathogenetic effect of the variant, but also support its dominant inheritance.

## Introduction

The ISCU gene (12q23.3; HGNC ID: 29882) is composed of 5 exons (983 nucleotides; NM_213595.4) and encodes a 15 kDa protein of 167 amino acid residues (NP_998760.1) performing a scaffolding function during iron-sulfur clusters assembly. Alternative splicing results in transcript variants encoding different protein isoforms that localize either to the cytosol (isoform 1 NM_014301.4) or to the mitochondrion (isoform 2 NM_213595.4) (Tong and Rouault, 2000). Isoform 1 contains an alternate exon in the 5′region and initiates translation at an alternative start codon compared to variant 2. Therefore, the cytosolic isoform has a distinct, shorter N-terminus. Mitochondrial isoform 2 encodes the longest transcript.

The ISCU structure consists of four α-helices which arc around the three antiparallel β-strands to form a planar platform for the molecule (Campbell et al., 2021). The active site, containing three conserved cysteine residues (Cys69, Cys95 and Cys138) and an aspartic acid residue (Asp71) (Fox et al., 2019), provides the setting where sulfur is delivered as a persulfide by cysteine desulfurase (NFS1) with the assistance of frataxin (FXN). Once iron is delivered, both substrates are used for Fe–S cluster assembly (Fox et al., 2019). Fe-S clusters are fundamental to numerous biological processes. They play a role in the functioning of a diverse set of enzymes, including those that regulate metabolism, iron homeostasis, DNA synthesis and repair, ribosome biogenesis, and oxidative stress response (Kollberg et al., 2009; Legati et al., 2017). Inability to express a full-length functional ISCU in humans results in Fe–S cluster deficiency, emphasizing the scaffold’s importance in relation to the cluster assembly pathway (Campbell et al., 2021).

Pathogenic/likely pathogenic variants in the *ISCU* have been linked to mitochondrial and neuromuscular disorders, manifesting in a variety of phenotypes. Hereditary myopathy with lactic acidosis due to ISCU deficiency is a rare disorder (MIM #255125) of energy metabolism characterized clinically by myopathy with severe exercise intolerance and biochemically by deficiencies of skeletal muscle mitochondrial respiratory chain enzymes, succinate dehydrogenase and aconitase. This mitochondrial myopathy is classically characterized by lifelong exercise intolerance in which minor exertion causes tachycardia, shortness of breath, fatigue and pain in active muscles. Episodes of more profound exercise intolerance are associated with rhabdomyolysis, myoglobinuria, and weakness. Affected individuals can have calves hypertrophy (Larsson et al., 1964; Haller et al., 1991; Kollberg et al., 2009; Kollberg et al., 2011).

So far, this disease has been molecularly identified in few patients: Swedish patients, homozygous for a deep intronic splicing affecting mutation in the *ISCU* (c.418 + 382G>C, also known as IVS5+382G>C, reported in ClinVar - a public archive of reports of human variations classified for diseases with supporting evidence - as a pathogenic variant) (Mochel et al., 2008; Olsson et al., 2008); a single Scandinavian family with a missense change in compound heterozygosity with the common intronic variant (c.418 + 382G>C and c.149G>A, p.Gly50Glu (reported in ClinVar as a likely pathogenic variant) (Kollberg et al., 2009) and an Italian patient heterozygous for the *de novo* dominant *ISCU* variant (c.287G>T, p.Gly96Val, not reported in ClinVar) (Legati et al., 2017). A disease-associated mutation in an intron (c.418 + 382G>C) activates a cryptic splice site, resulting in the production of a splice variant encoding a putatively non-functional protein (Nordin et al., 2011). Homozygosity for the common pathogenic splice site variant results in a mitochondrial disorder restricted to skeletal muscle with characteristic features of severe exercise intolerance. In Sweden, the carrier rate has been estimated at 1:188 (Mochel et al., 2008). Although data is limited, reported individuals, who are compound heterozygotes for the common pathogenic splice site variant and a missense variant, have had a more severe muscle phenotype with weakness and cardiomyopathy (Kollberg et al., 2009).

More recently, a heterozygous, *de novo* dominant *ISCU* variant (c.287G>T, p.Gly96Val) was reported in a patient with isolated myopathy and moderately high creatinine kinase (CK) and no involvement of other organs (Legati et al., 2017). Clinical, histological, histochemical and biochemical abnormalities affecting skeletal muscle were similar to those reported for *ISCU* recessive pathogenic/likely pathogenic variants, including a partial depletion of SDH and COX histochemical reactions, a generalised reduction of the mitochondrial respiratory chain complex activities, and the accumulation of iron deposits. Moreover, analysis of the corresponding genetic defect in yeast suggested a Fe-S protein biogenesis defect, including defects in OXPHOS and in cellular iron regulation. The effects were dominant, since both the monoallelic and the heteroallelic genotypes were associated with the phenotype (Legati et al., 2017).

In this paper, we report a case of a patient with progressive muscle weakness carrying a novel heterozygous variant in the *ISCU*. Presence of other exonic or deep intronic variants was excluded. Although the phenotype is reminiscent of a recessive disease, studies performed in yeast *Saccharomyces cerevisiae* not only confirm a pathogenetic effect of the variant but also support its dominant inheritance.

## Methods

### Genetic studies

Whole exome sequencing (WES) was performed on the proband DNA (obtained from peripheral blood) using Twist Human Core Exome Plus Kit (Twist Bioscience) and sequenced with Illumina technology (100x depth of mean coverage). Reads were aligned to the hg38 reference genome. Obtained QC value was <99% for Q30. Alignment and variant calling were performed with an in-house bioinformatics pipeline. Identified variants were annotated using the Ensembl VEP as well as multiple databases, including dbSNP, dbNSFP, gnomAD, ClinVar, and HGMD. XHMMv1.0 algorithm and in-house scripts were used to search for small, rare CNVs. American College of Medical Genetics and Genomics (ACMG) Standards and Guidelines for the interpretation of sequence variants were followed in this study. Standard Sanger sequencing was performed to confirm the presence of the identified variant in the proband and to assess segregation in the family.

### Yeast studies

The yeast strain used in this work, derived from W303-1B (*Matα ade2-1 leu2-3, ura3-1 trp1-1 his3-11, 15 can1-100*), is deleted at both *ISU1* and *ISU2* loci and expresses a wild type copy of *ISU1* gene cloned in the *URA3* bearing vector pFL38 to allow viability (Legati et al., 2017). Synthetic complete medium (SC) [0.69% yeast nitrogen base without amino acids (FormediumTM, UK)] supplemented with 1 g/L drop-out mix according to Kaiser et al. (1994), except amino acids and bases necessary to keep plasmids, was used to grow yeast strains. Media were supplemented with various carbon sources as indicated (Carlo Erba Reagents, Milan, Italy) in liquid phase or after solidification with 20 g/L agar (FormediumTM, UK). The human valine 134 residue corresponds to valine 135 in the yeast protein. The *isu1* ^V135*^ mutant allele was generated by PCR QuikChange™ (Agilent) using KOD Hot Start DNA Polymerase (Merck), as template DNA the *ISU1* cloned in the pFL39 plasmid (Legati et al., 2017) and the modified primers (base changes in bold) as follows: Isu1^V135*^Fw 5′-GGA​GTT​GAG​CTT​GCC​CCC​ATA​AAA​GTT​GCA​TTG​CTC​TAT​GTT​AG-3′ and Isu1^V135*^Rv 5′-CTA​ACA​TAG​AGC​AAT​GCA​ACT​TTT​ATG​GGG​GCA​AGC​TCA​ACT​CC-3’. After mutagenesis, sequence of the insert was verified by Sanger sequencing, and the pFL39*isu1* ^V135*^ was used to transform the *isu1*∆*isu2*∆/pFL38*ISU1* strain, using the lithium acetate method (Gietz and Schiestl, 2007). Transformants were selected for the presence of both constructs on solid SC medium lacking tryptophan and uracil. Yeast viability of *isu1*∆*isu2*∆ expressing *isu1* ^V135*^ was evaluated by analysing the ability to grow on SC medium supplemented with 1 mg/mL 5- fluoroorotic acid (5-FOA) monohydrate (ForMedium) and 2% glucose as described in Cappuccio et al. (2021). Briefly, viability assays were performed by growing cells of two independent clones in liquid SC medium supplemented with uracil for 24 h until the early stationary phase to induce the loss of the plasmid carrying the wt*ISU1*, and plating them (10-fold dilution spots starting from 4 × 10^5^ or 4 × 10^4^ cells/spot) in SC medium supplemented with 2% glucose, with or without 5-FOA. Results were scored after 72 h at 28°C.

For oxidative growth analysis, strains were serially diluted and spotted on SC-W agar plates, supplemented with 2% glycerol or 2% lactate or 2% glucose. The plates were incubated at 28°C. Cell yield was determined by growing cells on liquid medium containing glucose or glycerol and measuring the optical density at 600 nm after 72 h of growth at 28°C. Oxygen consumption rate (OCR) was measured by a Clark-type oxygen electrode (Oxygraph System Hansatech Instruments) (Figuccia et al., 2024) on whole cells grown in SC-W medium supplemented with 0.5% glucose until exhaustion for 18 h at 28°C.

## Results

### Clinical presentation

A 59-year-old patient (II:2) was referred to a Genetic Clinic (Institute of Psychiatry and Neurology in Warsaw, Poland) due to progressive weakness of hip and shoulder girdle muscles, which appeared around the age of 34. Physical examination revealed abnormal, unsteady gait, positive Gower`s sign, weakened tendon reflexes, great difficulty climbing and descending stairs. Periodic CK levels were normal. Muscle biopsy reported: “Small, uncharacteristic changes: centrally located nuclei, myofibril defects, one circular fiber”. In the electrocardiogram (ECG) examination - sinus rhythm, regular, normogram. Tandem MS/MS, lactic acid level in peripheral blood - no metabolic defects were found. Electromyography (EMG) has not been performed. Family history was negative. Limb girdle muscular dystrophy (LGMD) was suspected, and the patient underwent genetic testing. Myotonic dystrophy type 2 (MIM #602668) and a common pathogenic variant: c.550delA (NM_000070.3) in the *CAPN3* gene were excluded.

### Genetic studies

**FIGURE 1:**
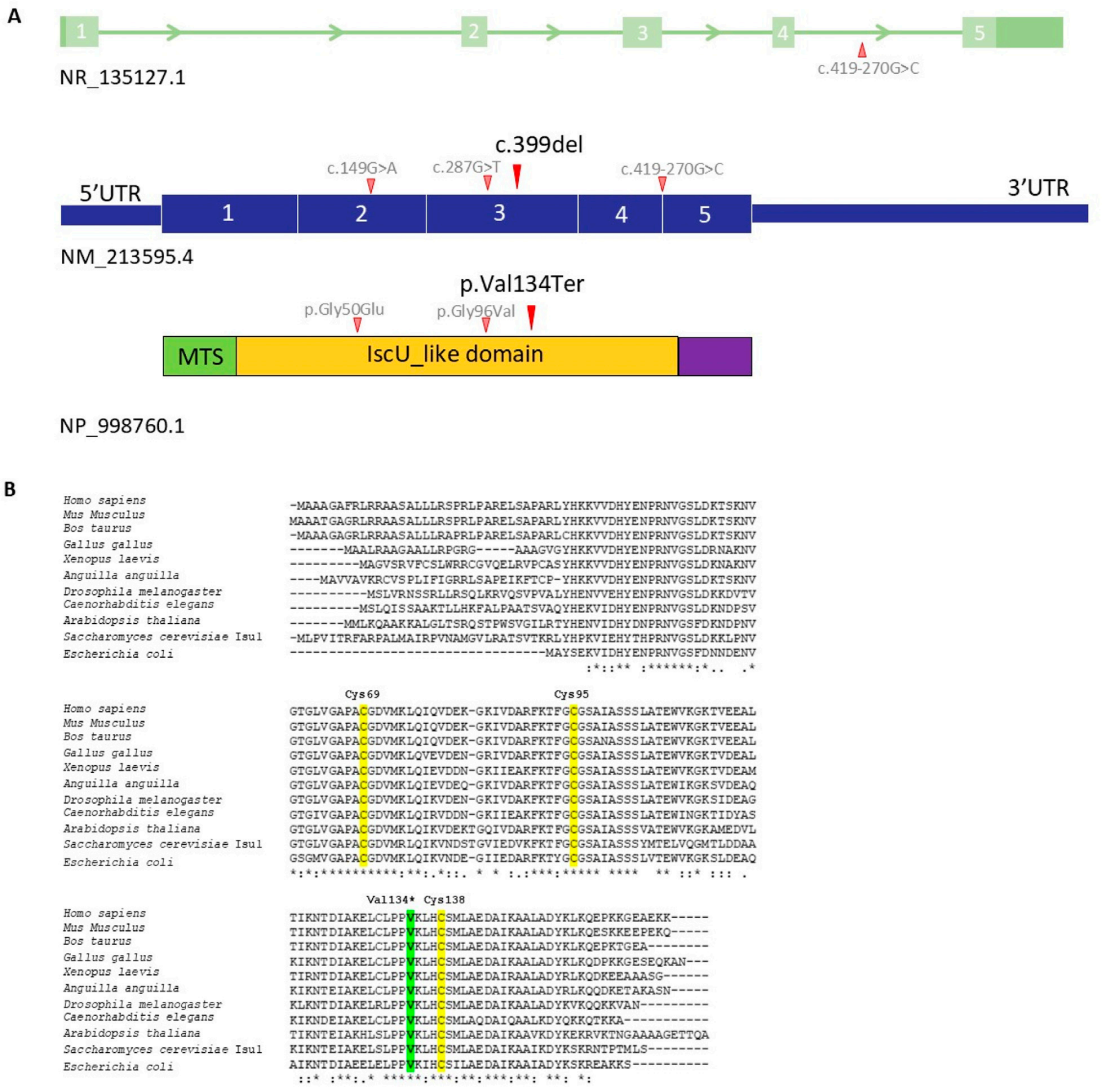
**(A)** Schematic representation of the ISCU gene, cDNA (NM_213595.4) and protein with the nucleotide/amino acid change identified in the Family (black) and literature (grey). The functional IscU-like domain is in yellow; the mitochondrial targeting sequence (MTS) is in green. **(B)** Multi-organism alignment. Human ISCU was aligned with ISC proteins from various organisms, including mammals, birds, amphibians, bony fishes, arthropods, nematodes, dicotyledon, fungi, and prokaryotes. The residue affect by mutation is highlighted in green, while Cys69, Cys95, and Cys138 which from part of the active site, are shown in yellow.

WES indicated a novel heterozygous variant c.399del (p.Val134Ter, exon 4) in the *ISCU* gene. Presence of a second exonic variant or deep intronic variants (including c.418 + 382 or IVS5+382G>C variant) were excluded. Presence of the c.399del variant was confirmed by Sanger sequencing in the proband (Supplementary Figure S1). No copy number variants (CNVs) were identified. The variant allele was not found in control chromosomes in the gnomAD (v3 and v4) database. The variant causes a premature termination of the protein. The variant is localized in the functional IscU_like domain of the ISCU protein (Figure 1A) with bioinformatics prediction of damaging effects on protein function. The variant affected a highly conserved residue throughout evolution (Figure 1B) and gave high scores of pathogenicity, according to several bioinformatics tools.

According to the ACMG guidelines (Richards et al., 2015), the c.399del (p.Val134Ter) in the *ISCU* gene is categorized to be “likely pathogenic variant”, because it belongs to PP1 (cosegregation with disease in multiple affected family members in a gene definitively known to cause the disease), PPP4 (patient’s phenotype and family history is highly specific for a disease with a single genetic etiology), PS3 (functional studies show damaging effect on the gene or gene product), PM2 (extremely low frequency in gnomAD population databases), and PM4 BP3 (protein length changes due to in-frame deletions/insertions and stop losses).

**FIGURE 2:**
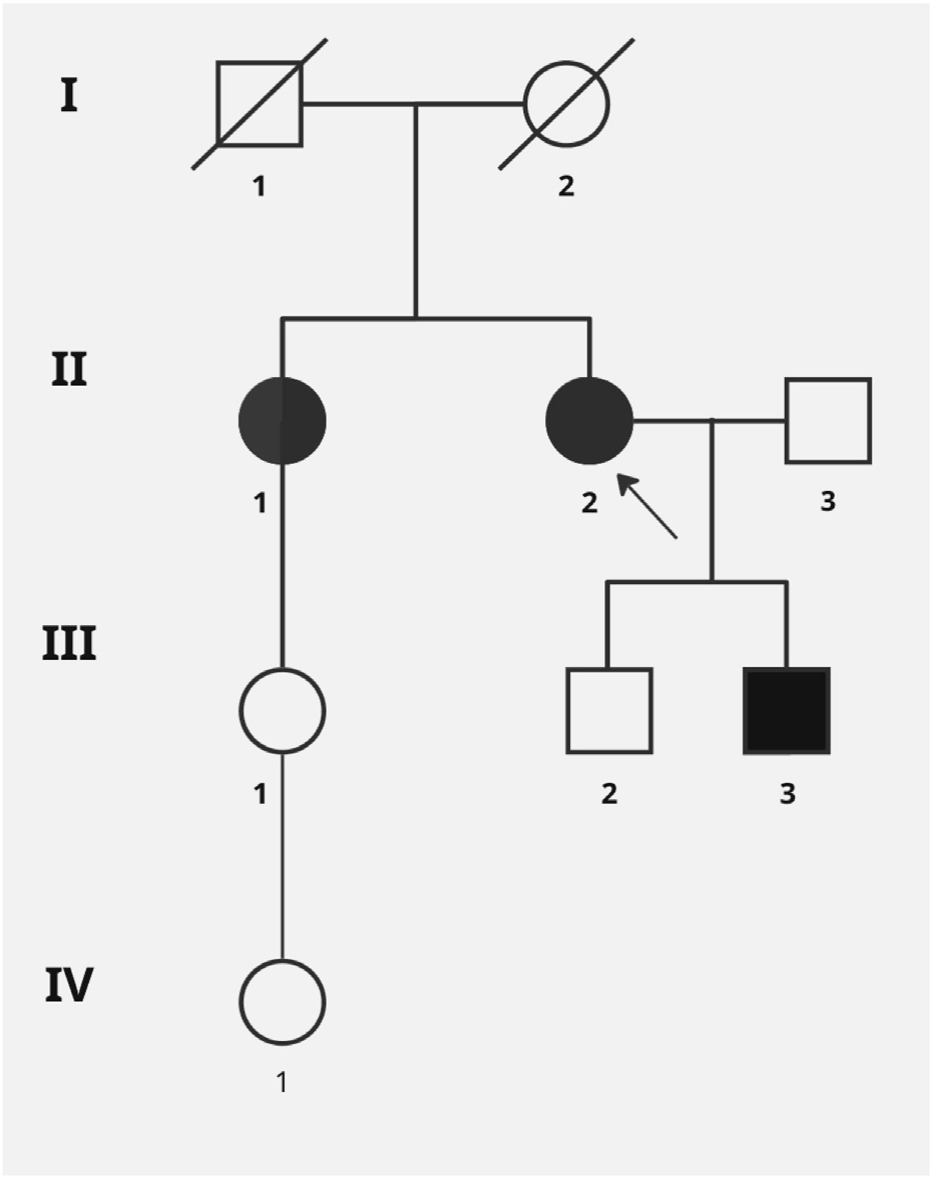
Pedigree of the reported family. Black symbols designate affected subjects, and the arrow point to the index case. Genetic test (Sanger sequencing) for c.399del variant was performed for all family members except I:1, I:2 (DNA sample not available) and II:3.

Clinical examination and genetics tests for the proband’s family were carried out (Figure 2 Pedigree).

Son 1 (III:2) was referred to a genetic clinic at the age of 37. Physical examination: no gait disturbances or significant muscle weakness. CK level–twice normal. Lactate level normal. He did not consent to the EMG examination. No familial variant in the ISCU was found.

Son 2 (III:3) was referred to a genetic clinic at the age of 32. Physical examination: no gait disturbances or significant muscle weakness, slight calves hypertrophy. CK level slightly elevated: 204.4 U/I (<190) and 376.2 U/L (<190.0), lactate level (peripheral blood) normal. He did not consent to the EMG examination. The presence of a familial variant: p.Val134Ter in the *ISCU* was confirmed.

The proband’s sister (II:1) was referred to a genetic clinic at the age of 77. Assessment of the musculoskeletal system during physical examination was difficult, the patient was bedbound, in the terminal stage of cancer. According to interview data, before the diagnosis of cancer, she walked with a cane and reported difficulties with walking since early adulthood. CK level, lactates, EMG were not performed due to lack of consent. The presence of the p.Val134Ter variant in the *ISCU* was confirmed.

Daughter of the sister of proband (III:1) was referred to a genetic clinic at the age of 58. Physical examination: no gait disturbances, no muscle weakness. The level of CK and lactates in peripheral blood was normal. No pathogenic variant was found in the *ISCU*.

Daughter of the daughter proband’s first sister (IV:1) was referred to a genetic clinic at the age of 37. Physical examination: no gait disturbances, no muscle weakness. The level of CK and lactates in peripheral blood was normal. No pathogenic variant was found in the *ISCU*.

In Table 1 resumed the clinical features of all patients described with *ISCU* variants.

**TABLE 1:** Clinical characteristics of patients with *ISCU* variants.

Patient	II:2	III:3	II:1	Patient described by Legati et al. (2017)	Patients summarized by Kollberg et al. (2009)
Sex	F	M	F	M	F and M
Age at examination	59	32	77	23	Childhood
Disease onset	Early adulthood	Adulthood (?)	Early adulthood	Childhood	Childhood
Muscle weakness	+	−/+	+	+	+/−
Exercise intolerance	+	+	+	+	+
Gait	Waddling gait; unable to run	Normal	Waddling gait; unable to run	Able to walk; unable to run	Waddling gait; unable to run
Other physical findings	Positive Gower’s sign; weakened tendon reflexes; great difficulty climbing and descending stairs	Slight calf hypertrophy	Walking with cane (prior to cancer diagnosis)	Distal limb weakness with muscle atrophy; absent deep tendon reflexes	Early fatigue on exertion, dyspnea, palpitations; muscle cramps and weakness with sustained activity
Creatine kinase (CK)	Normal	Slightly elevated	NA	Elevated	Elevated
Lactate	Normal	Normal	NA	Elevated	Elevated
ECG	Normal	NA	NA*	Normal	Normal
Mitochondrial enzymes	NA	NA	NA	Decreased	Decreased
*ISCU* pathogenic variant	Heterozygous c.399del (p.Val134Ter)	Heterozygous c.399del (p.Val134Ter)	Heterozygous c.399del (p.Val134Ter)	Heterozygous c.287G>T (p.Gly96Val)	Homozygous c.418 + 382G>C or compound heterozygous c.418 + 382G>C and c.149G>A (p.Gly50Glu)
Inheritance pattern	Dominant	Dominant	Dominant	Dominant	Recessive
Disease course	Progressive	(?)	Progressive*	Progressive	Progressive
Symptom severity	Mild to moderate	NA	Mild*	Moderate	Severe

Abbreviations: F, female; M, male; NA, Not available; “+”, Present; “-”, Absent; “*”, Difficult to assess due to complex phenotype.

### Functional studies in yeast

**FIGURE 3:**
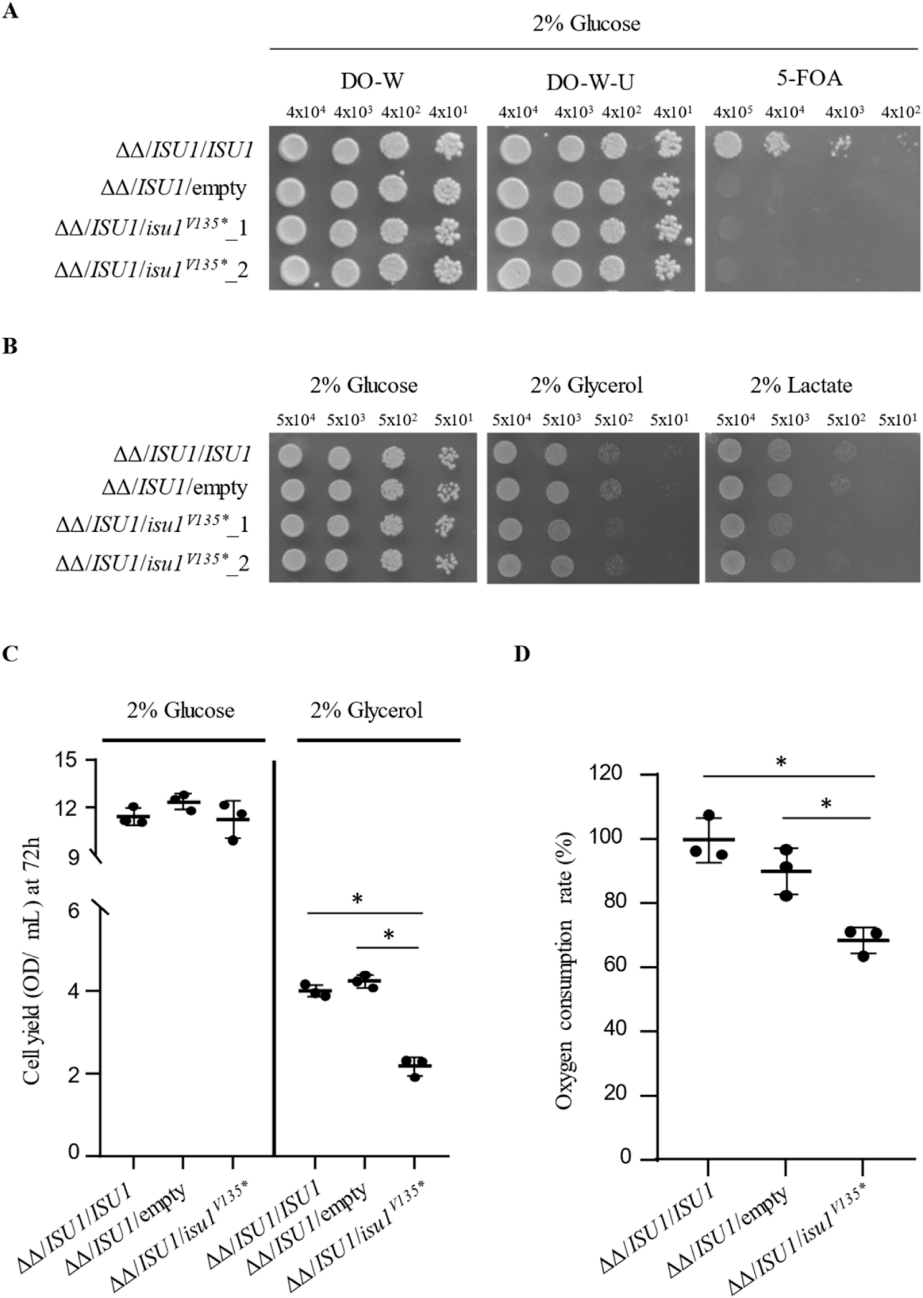
Yeast functional analysis **(A)** Viability of *isu1Δisu2Δ* strains harbouring pFL38/*ISU1* and transformed with *wtISU1*, or *isu1* ^V135*^ mutant allele, cloned in pFL39, or pFL39 empty vector was evaluated by growth assays. Cell were grown on SC medium lacking tryptophan or both uracil and tryptophan, supplemented with 2% glucose, with or without 0.1% 5-FOA, at 28°C.Pictures were taken after 72 h. **(B)** Oxidative growth phenotype of the same strains as in **(A)** was evaluated in SC-U-W medium supplemented with the indicated carbon sources at 28°C. Ten-fold dilutions starting from 5 × 10^4^ cells/spot were spotted. Pictures were taken after 48 h. **(C)** Cell yield of the same strains described in **(A)** was calculated by growing cells on liquid SC-U-W medium containing glucose or glycerol at 28°C and measuring the optical density at 600 nm after 72 h. **(D)** Oxygen consumption rate (OCR) was carried out on the strains reported above grown in liquid SC-U-W medium supplemented with 0,5% of glucose at 28°C. Values are means ± standard deviation. *: p < 0.05 in One-Way ANOVA test with Bonferroni correction.

To assess the pathogenicity of the novel p.Val134Ter *ISCU* variant, we performed complementation studies in *Saccharomyces cerevisiae* strain lacking the orthologous gene *ISU1* and the paralogue *ISU2*, hereafter referred to as *isu1*Δ*isu2*Δ, harbouring the centromeric pFL38 plasmid (*URA3* marker) with the wild type (wt) *ISU1* to allow viability (Legati et al., 2017). Since the p.Val134 residue is conserved between the two species, corresponding to p.Val135 in yeast, the change, equivalent to the human variant, was directly introduced into the yeast *ISU1* wild type. The strain *isu1*Δ*isu2*Δ/*ISU1* was transformed with pFL39 centromeric plasmid (*TRP1* marker) containing either the mutant allele *isu1* ^V135*^, a wt copy of *ISU1*, or no gene and the viability of the transformed strains was tested in medium supplemented with 5-FOA on which only cells which have lost wt *ISU1* could grow. As shown in Figure 3A, the *isu1* ^Val135*^ allele was unable to rescue the lethal phenotype of *isu1*Δ*isu2*Δ since no growth was observed in 5-FOA indicating that is*u1* ^Val135*^behaves as *null* allele and confirming the pathogenicity of this variant.

Yeast not only represents an excellent model system for investigating the role of novel variants in mitochondrial-related conditions but also a flexible tool also to assess the dominant or recessive nature of a mutation (Ceccatelli Berti et al., 2021). We then evaluated whether the Val135* mutation could act as a dominant trait. To this end we compared the growth phenotype of the heteroallelic strain *isu1*Δ*isu2*Δ/*ISU1*/*isu1* ^V135*^ with that of the homoallelic strain *isu1*Δ*isu2*Δ/*ISU1*/*ISU1* and the *isu1*Δ*isu2*Δ/*ISU1*/hemiallelic one on both fermentable and non fermentable carbon sources. As shown in Figure 3A, on glucose containing medium no difference in fermentative growth was observed between the strains analysed. However, on medium containing glycerol or lactate, a condition in which the mitochondrial functionality is essential to obtain ATP, the growth of the heteroallelic strains shows a reduction, although slight, not only respect to that of the homoallelic strain, but also to that of the hemiallelic strain (Figure 3B).

In addition to the spot assay, to precisely and quantitatively evaluate the growth phenotype of mutant strains, not detectable with a semi-quantitative test such as a spot assay, a liquid-phase growth assay could be performed allowing the identification of minor defects. For this reason the mild reduction of oxidative growth displayed by the heteroallelic strain *isu1*Δ*isu2*Δ/*ISU1*/*isu1* ^V135*^ was better investigated by measuring the cell yield of the aforementioned strains in liquid medium. The result obtained confirmed what was observed in the previous analysis since the reduction was approximately 50% compared to both control strains (Figure 3C). Finally, oxygen consumption rate (OCR) can be measured to allow to determine the severity of the damage. Consistently, OCR was reduced by about 30% in the heteroallelic strain *isu1*Δ*isu2*Δ/*ISU1*/*isu1* ^V135*^ respect to both homoallelic and hemiallelic strain (Figure 3D). Together these results indicates that the Val135* mutation is dominant.

## Discussion

Hereditary myopathy with lactic acidosis due to ISCU deficiency, also known as Swedish-type myopathy with exercise intolerance, is described as an autosomal recessive disorder. However, in this study, we presented the first family in which an autosomal dominant pattern of inheritance of *ISCU*-related myopathy has been identified. So far, only one sporadic case of *ISCU*-related myopathy caused by a mutation in a single allele of this gene has been described (Legati et al., 2017).

In the diagnostic process of our family, we used exome sequencing, which included CNV analysis and covered deep intron regions where known pathogenic variants are located. We identified a novel *ISCU* terminating variant c.399del in the exon 4, according to the MANE sequence. This change leads to the premature stop codon (p.Val134Ter) resulting in production of a shortened protein that is likely non-functional or/and possibly activates nonsense-mediated decay (NMD). Moreover, premature termination leads to the removal of one of three conserved cysteine residues (Cys183) in the active site of the enzyme, which could significantly impact protein function (Fox et al., 2019). It is known that the inability to express a full-length ISCU in humans results in Fe–S cluster deficiency (Tong and Rouault, 2000, Mochel et al., 2008; Tong and Rouault, 2006; Kollberg and Holme, 2009, Rawcliffe et al., 2018; Campbell et al., 2021). Furthermore, functional analysis has shown that suppression of human ISCU by RNAi not only inactivates mitochondrial and cytosolic aconitases, but also inappropriately activates the iron regulatory proteins thus disrupting intracellular iron homeostasis (Tiong and Rouault, 2006).

One of the best characterized *ISCU* variants is the deep intronic c.418 + 382G>C splice site mutation, detected in patients from northern Sweden. This change affects mRNA splicing and gives rise to the insertion of a pseudoexon between the last two exons in the mRNA sequence and to the introduction of a premature stop codon in the penultimate exon. This ultimately alters the C terminus of the protein and decreases levels of ISCU mRNA and protein in patients’ fibroblasts and lysates from skeletal muscle mitochondria (Mochel et al., 2008; Kollberg and Holme, 2009; Kollberg et al., 2009; Rawcliffe et al., 2018). Since the variant detected in our family is located in the penultimate exon, we suspect that it could have a similar effect.

Genetic segregation analysis and functional studies performed in the yeast *Saccharomyces cerevisiae* as a model system supported the dominant effect of the new identified variant and confirmed its gene–disease relationship. In particular, pedigree and clinical analyses have shown that the variant detected in the present work segregates with the symptoms of myopathy in various family members. Both our proband and her sister had great difficulty walking. One of the proband’s sons demonstrated mild calf enlargement, slightly elevated CK levels, and initial symptoms suggestive of developing myopathy. No increase in blood lactate levels were observed. Moreover, the symptoms of our proband were slightly different from those of the patient described by Legati et al., who mainly had distal muscle weakness, and whose phenotype was more reminiscent of the recessive disease. Our patient suffered in the first line from proximal muscle weakness, with periods of significant deterioration, and had a LGMD-like phenotype. Furthermore, CK level in our family ranged from normal to slightly elevated and no rhabdomyolysis was observed in any of them (Angelini, 1993). Due to the lack of EMG testing and muscle biopsy combined with biochemical tests in all family members, the myopathy phenotype is not fully defined. The further evaluation of the muscle biopsy was not possible and the other family members for the segregation were not available. Therefore, further clinical observation over time is required to better understand the progression of the disease.

In order to confirm the pathogenicity of the novel p.Val134Ter *ISCU* variant and to assess the dominant or recessive nature of this mutation we take advantage of the yeast ISCU model, i.e., a *S. cerevisiae* strain deleted in both *ISU1*and *ISU2* genes and expressing the variant allele *isu1* ^V135*^and a wt copy of *ISU1*. The mutant allele was unable to complement the lethal phenotype of the null *isu1*Δ*isu2*Δ double mutant thus confirming the complete loss of function of the truncated protein. Moreover, the oxidative growth phenotype of the heteroallelic strain *isu1*Δ*isu2*Δ/*ISU1*/*isu1* ^V135*^ has shown a slight defect referred to both the homoallelic and hemiallelic strains. This impairment of the oxidative metabolism was supported by the reduced respiratory rate in the ISCU model. These results indicate that the Val135Ter mutation is dominant and, since the phenotype of the heterozygous strain is more severe than that of the hemizygous strain, one can speculate that the mutation alone can be the cause of the pathology due to a negative dominance.

In the context of neuromuscular diseases, both recessive and dominant inheritance patterns for the same disease have been reported, with variable severity of clinical phenotypes within the families for the dominant variants, as seen in some LGMDs, such as calpainopathies (Angelini, 1993) or dysferlinopathies (Folland et al., 2022). It is known, also for mitochondrial diseases, that both recessive and dominant mutations in the same gene may lead to the same disease (Legati et al., 2017).

To date, only two pathogenic *ISCU* variants are reported in ClinVar: c.149G>A (p.Gly50Glu) located in the exon 4 and c.418 + 382G>C located in the intron region between exons 4 and 5. Approximately 60 variants are classified as variants of unknown significance (VUS). Some of them are associated with hereditary myopathy with lactic acidosis, however, functional evidence in ClinVar is missing for many of these variants.

This underscores the need for functional studies to better understand and characterize the effect of novel variants on protein function and metabolic pathways. In this regard, as this study also shows, yeast model once more proves to be a useful tool that can be used to assess the impact of VUS in a quick and efficient way (Ceccatelli Berti et al., 2021; Figuccia et al., 2024).

Our study confirms that a deep analysis of WES data is essential in the diagnosis process of genetic diseases. It should assume any mode of inheritance for genes directly related to the phenotype and/or condition. Although no specific therapy currently exists for ISCU-related myopathy, available evidence suggests that the disease correlates with a relatively normal lifespan. The disease has a chronic and progressive course with exacerbations and remissions. Further clinical and functional studies are needed to better understand the molecular mechanism and progression of the disease and to identify potential therapeutic approaches.

## Data Availability

Due to ethical and privacy considerations related to this brief study report, in which the analysis was based on WES data from a single patient followed by family analysis and functional studies, the datasets generated and/or analyzed during the current study (including VCF and BAM files) are not publicly available. However, they are available from the corresponding author upon reasonable request, provided that the request complies with applicable ethical approvals and data protection regulations.

## Supplementary material

Supplementary Figure 1 Electropherograms of the *ISCU* fragment showing the c.399del variant [NM_213595.4] in family members. The proband (II:2), the affected sibling (II:1), and the affected son (III:3) display a heterozygous variant (arrow), while the unaffected son (III:2) presents a wild-type sequence.
