# Supplementary figures and images for: Autosomal dominant myopathy caused by a novel ISCU variant
Source: Front Genet. 2025 Jun 2;16:1605440. doi: 10.3389/fgene.2025.1605440 (PMC12171185; doi:10.3389/fgene.2025.1605440)

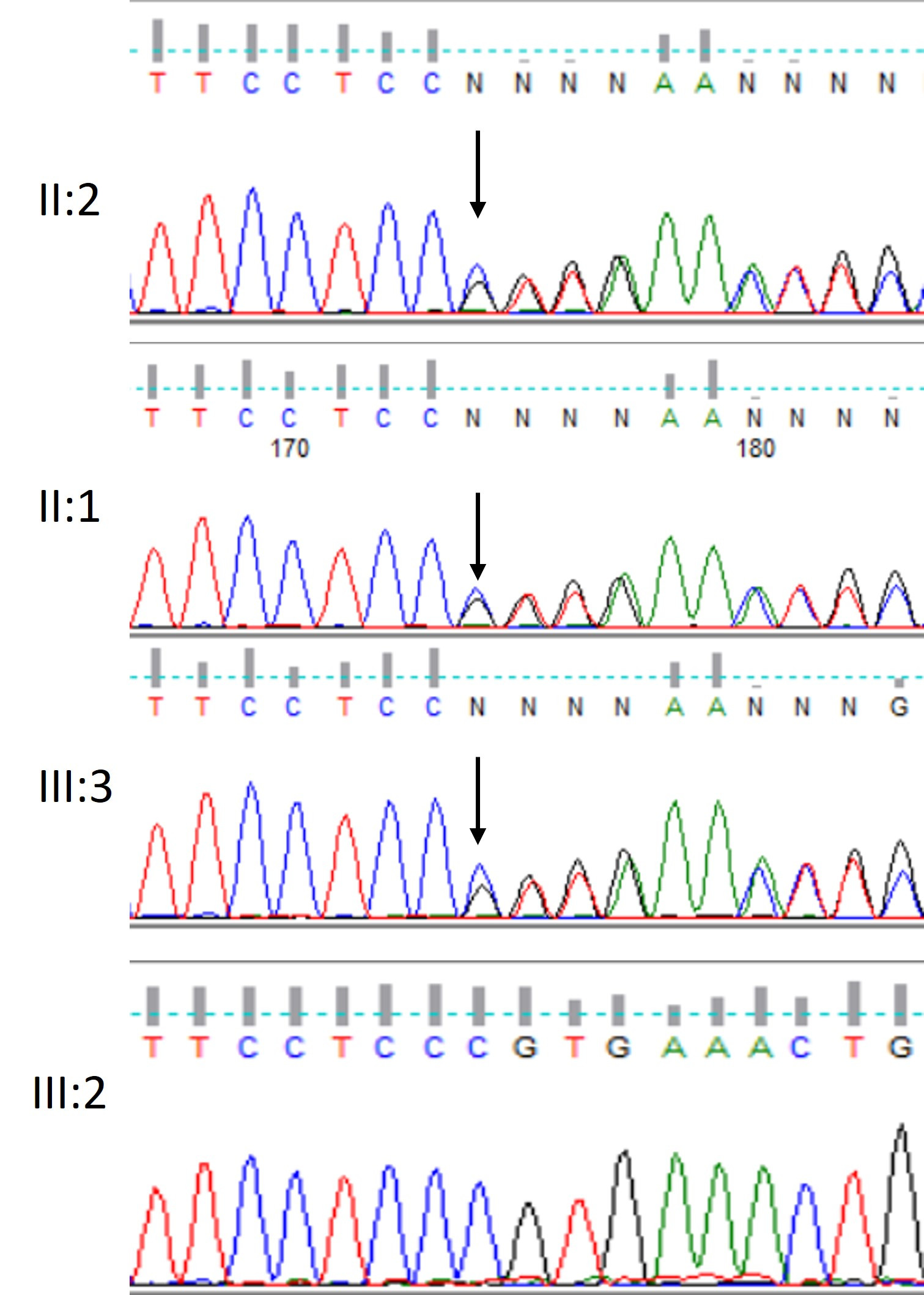

Supplement: Supplementary file 1 [file Image1.jpeg]
